# Table for five, please: Dietary partitioning in boreal bats

**DOI:** 10.1002/ece3.4559

**Published:** 2018-10-12

**Authors:** Eero J. Vesterinen, Anna I. E. Puisto, Anna S. Blomberg, Thomas M. Lilley

**Affiliations:** ^1^ Biodiversity Unit University of Turku Turku Finland; ^2^ Department of Agricultural Sciences University of Helsinki Helsinki Finland; ^3^ Department of Biology University of Turku Turku Finland; ^4^ Institute of Integrative Biology University of Liverpool Liverpool UK; ^5^ Finnish Museum of Natural History University of Helsinki Helsinki Finland

**Keywords:** Chiroptera, dietary analysis, metabarcoding, prey size, resource partitioning

## Abstract

Differences in diet can explain resource partitioning in apparently similar, sympatric species. Here, we analyzed 1,252 fecal droppings from five species (*Eptesicus nilssonii, Myotis brandtii, M. daubentonii, M. mystacinus*, and *Plecotus auritus*) to reveal their dietary niches using fecal DNA metabarcoding. We identified nearly 550 prey species in 13 arthropod orders. Two main orders (Diptera and Lepidoptera) formed the majority of the diet for all species, constituting roughly 80%–90% of the diet. All five species had different dietary assemblages. We also found significant differences in the size of prey species between the bat species. Our results on diet composition remain mostly unchanged when using either read counts as a proxy for quantitative diet or presence–absence data, indicating a strong biological pattern. We conclude that although bats share major components in their ecology (nocturnal life style, insectivory, and echolocation), species differ in feeding behavior, suggesting bats may have distinctive evolutionary strategies. Diet analysis helps illuminate life history traits of various species, adding to sparse ecological knowledge, which can be utilized in conservation planning.

## 1 INTRODUCTION

Coexistence of sympatric species is facilitated by differences in the use of resources, that is, resource partitioning (Schoener, 1974). Resource partitioning occurs in several dimensions, with regard to resources. Ultimately, the sum of these dimensions constitutes the ecological niche of an organism, that is, the set of biotic and abiotic conditions in which a species can persist (Holt, 2009). This includes both the distribution of a species and its interactions with other species, but also factors relevant to the fine‐scale distribution of species (e.g., microhabitats), their biotic interactions as well as their diet (Wiens et al., 2010).

With a notable adaptive radiation in their evolutionary history, and over 1,300 known species worldwide (Fenton & Simmons, 2015), bats have an important role in supporting global ecosystems through their dietary preferences. This is evidenced primarily through the consumption of nocturnal insects and dispersal of nutrients, pollen, and seeds (Patterson, Willig, & Stevens, 2003). Research on the feeding behavior of species is essential to understanding ecosystem function and the impacts of pollution, habitat destruction, and global climate change (Boyles & Storm, 2007; Kunz, Braun de Torrez, Bauer, Lobova, & Fleming, 2011; Vesterinen, 2015; Vesterinen et al., 2016). Furthermore, establishing factors influencing the extinction risk of bats is essential for their conservation, because they help identify endangered species and provide the basis for conservation (Safi & Kerth, 2004). However, these factors may be difficult to discern between species of bats, of which many appear to share portions of their ecological niches, such as habitat and apparently diet.

Even though some degree of food mixing is required for most species, it is thought that the diets of terrestrial mammals are generally highly specialized (Pineda‐Munoz & Alroy, 2014). Indeed, when viewed in its entirety, the dietary diversity in bats is huge, ranging from insectivores, frugivores, and nectarivores to piscivores, carnivores, and even sanguinivores (Kunz, 1998). However, closely related species often occupy similar ecological niches, suggesting that components of the diet overlap to a high degree (Lara, Pérez, Castillo‐Guevara, & Serrano‐Meneses, 2015; Losos, 2008; Münkemüller, Boucher, Thuiller, & Lavergne, 2015; Razgour et al., 2011; Wilson, 2010). This phylogenetic signal in food webs is associated with the tendency of related species to share habitat and body size (Rezende, Albert, Fortuna, & Bascompte, 2009). For instance, insectivorous bats are generally small, because of the negative correlation between size and echolocation frequency of a bat. High‐frequency echolocation calls are needed for the detection of small prey (Brigham, 1991). Nevertheless, species with identical niches rarely exist (Wiens et al., 2010).

Consisting of ca. 430 species sharing similar morphology, the insectivorous family Vespertilionidae [Gray 1821] is a useful group for research on resource partitioning (Aldridge & Rautenbach, 1987; Saunders & Barclay, 1992). Vespertilionidae exhibits only subtle interspecific morphological variation compared to members of the other bat families, even among distantly related species. This has posed a challenge in elucidating their evolutionary history (Jones, Purvis, MacLarnon, Bininda‐Emonds, & Simmons, 2002; Van Den Bussche & Lack, 2013). Similarities in morphology are mirrored in diet; the almost cosmopolitan vesper bats are primarily insectivorous (Hoofer & Bussche, 2003; Simmons, 2005; Van Den Bussche & Lack, 2013). However, based on feeding behavior, vesper bat species have been classified to guilds of either aerial‐hawking, gleaning, or trawling bats according to their foraging behavior (Norberg & Rayner, 1987). Recent advances in molecular methodology have begun to offer a deeper insight into the cryptic diet of these animals (Roslin, Majaneva, & Clare, 2016; Vesterinen et al., 2016; Vesterinen, Lilley, Laine, & Wahlberg, 2013). Vesper bats within the same feeding guild appear to share a great proportion of their diet (Roswag, Becker, & Encarnação, 2018). Because insectivorous bats opportunistically consume prey that may be periodically abundant (Vesterinen et al., 2013), this leads to significant temporal changes in the diet (Vesterinen et al., 2016), but could additionally result in a large overlap in dietary niches, suggesting resource partitioning occurs in other ecological dimensions.

**Figure 1:**
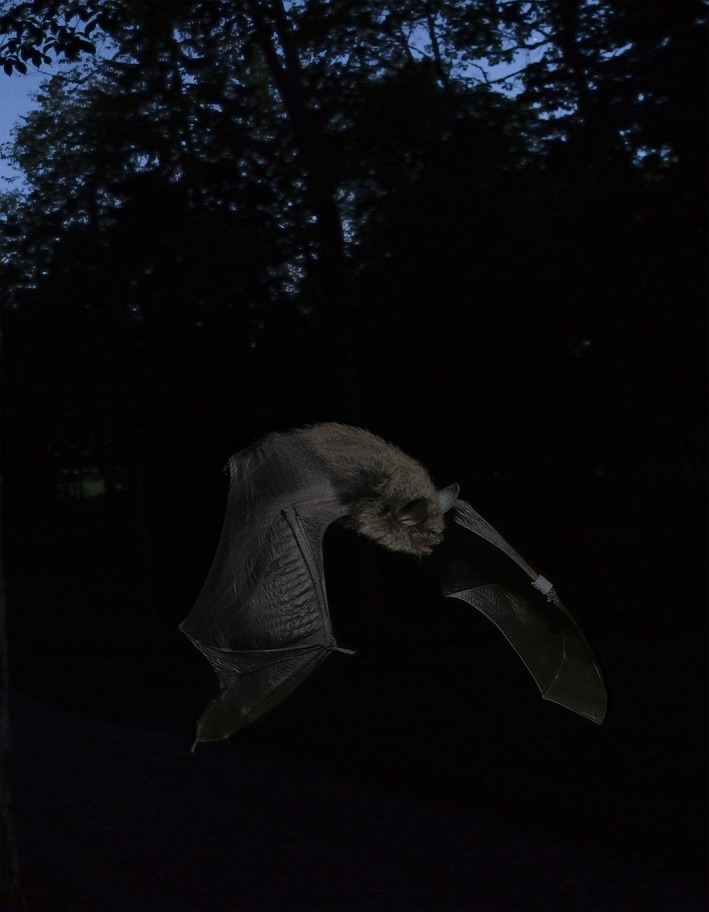
One of the study species, *Myotis brandtii*, foraging in its natural environment near the study area in southwestern Finland. *M. brandtii* catches its prey mainly in flight in an open or semi‐open environment. The current study is the first ever published molecular analysis of its diet: Geometrid and tortricid moths constituted half of its diet, while mosquitos, midges, and flies formed another large part of the menu, approximately one‐third. Photograph credits: Mr. Risto Lindstedt

Here, we unravel the resource partitioning of five resident vesper bats in southwestern Finland through deep dietary analysis, including prey species identification, an estimate for prey body size and temporal changes in diet using fecal DNA barcoding. At high northern latitudes, the distribution of bats is constrained by extreme environmental demands and prey availability is more seasonal than elsewhere in their range (Clare et al., 2014; Shively & Barboza, 2017; Shively, Barboza, Doak, & Jung, 2017). The ranges of these five species (*Eptesicus nilssonii* [Keyserling & Bläsius, 1839], *Myotis daubentonii* [Kuhl, 1817], *M. mystacinus* [Kuhl, 1817], *M. brandtii* [Eversmann, 1845], and *Plecotus auritus* [Linnaeus, 1758]) show considerable overlap, suggesting that trophic resource partitioning is important in supporting the species in Fennoscandia. We expect to see clear guild‐specific segregation in diet between the three different feeding guilds presented by our species, trawling (*M. daubentonii*), gleaning (*P. auritus*), and aerial hawking (Figure 1; *M. brandtii*,* M. mystacinus,* and *E. nilssonii*), and that we will see at least a partial dietary overlap among the members of the aerial hawkers. Because of the opportunistic foraging behavior of insectivorous bats (Vesterinen et al., 2013), we also predict significant temporal changes in diet throughout the sampling season (but see Vesterinen et al., 2016). Finally, we predict a positive correlation between predator and prey size, which could be due to the negative correlation between bat size and echolocation frequency, hindering the ability to detect small prey items (Brigham, 1991). To the best of our knowledge, of the species studied here, molecular data on diet exist only for *M. daubentonii* (Galan et al., 2018; Krüger, Clare, Greif, et al., 2014; Krüger, Clare, Symondson, Keišs, & Pētersons, 2014; Vesterinen et al., 2013, 2016 ), although the dietary contents of all species have previously been described through morphological analysis of fecal remains (Rydell, 1986; Vaughan, 1997).

## 2 MATERIALS AND METHODS

### 2.1 Study species

Of the 13 species of bats occurring in Finland, the species sampled here represent the most common and accessible (*Myotis daubentonii*,* Eptesicus nilssonii*,* M. brandtii*,* M. mystacinus*, and *Plecotus auritus*). Based on both the Finnish Biodiversity Information Facility (http://www.laji.fi) databases and our own bat sampling, spanning for more than 10 years, these bat species constitute approximately 90%–98% of all bat occurrences in Finland, and have been the focus of most bat research in Finland so far (Jakava‐Viljanen, Lilley, Kyheröinen, & Huovilainen, 2010; Laine, Lilley, Norrdahl, & Primmer, 2013; Lilley et al., 2013; Lilley, Stauffer, Kanerva, & Eeva, 2014; Lilley, Veikkolainen, & Pulliainen, 2015; Veikkolainen, Vesterinen, Lilley, & Pulliainen, 2014).

**Figure 2:**
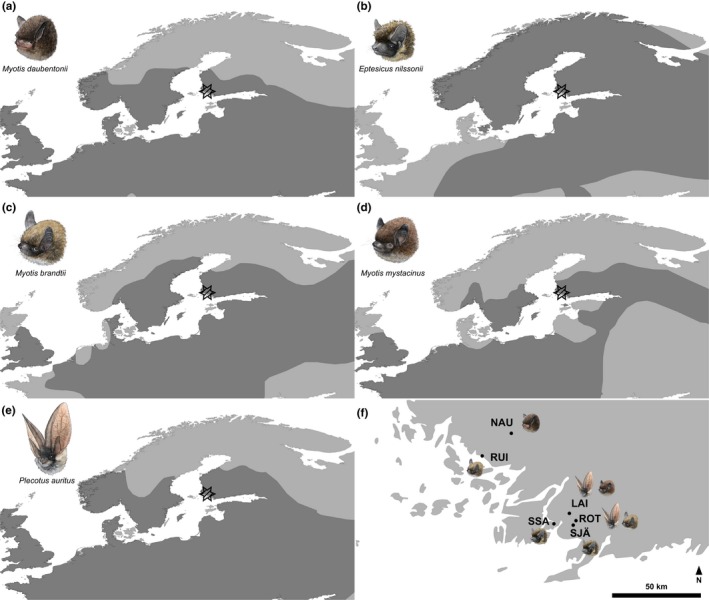
The map showing the distribution of each studied bat species in northeastern Eurasia: (a) *Myotis daubentonii*, (b) *Eptesicus nilssonii*, (c) *M. brandtii*, (d) *M. mystacinus*, and (e) *Plecotus auritus* with a star denoting the focal area of the current study. (f) Locations of the roost sites for each bat species in the current study in southwestern Finland: NAU = Nautelankoski (*M. daubentonii*), RUI = Ruissalo (*M. brandtii*), SJÄ = Sahajärvi (*E. nilssonii*), SSA = Särkisalo (*E. nilssonii*), LAI = Laiterla (*P. auritus* and *M. mystacinus*), and ROT = Rotholma (*P. auritus* and *M. brandtii*)

Of the sampled species, only the Northern bat (*Eptesicus nilssonii*) has a range encompassing all of Finland, with records extending far above the Arctic Circle, all the way to Utsjoki at 69°45′27, 27°1′29 (Figure 2b; Iso‐Iivari, 1988; IUCN, 2016a). Although records of *M. daubentonii* extend to the Arctic Circle (Figure 2a; IUCN, 2008a; Siivonen & Wermundsen, 2008), the distributions of most of the other focal species, *M. mystacinus*,* M. brandtii*, and *P. auritus*, are considered to reach their northern limits in central Finland (Figure 2c–e; IUCN, 2008b, 2008c, 2016b). These five species, with the addition of the extremely rare *M. nattererii* and *M. dasycneme*, are most likely the only regularly hibernating species in Finland, whereas the other species migrate or are infrequent visitors (but see Ijäs, Kahilainen, Vasko, & Lilley, 2017).

### 2.2 Field sampling

Fecal pellets were collected between April and July 2014 (Table 1) from day roosts of five species of bats in southwestern Finland, and all these roosts were in buildings within approximately 60 km of each other (Figure 2f). The pellets were collected by placing a clean paper sheet under the roosting bats the day before the collection, and collecting the droppings the next day. The collection was repeated for two or three consecutive days within a period of two weeks. Pellets were stored in RNA later at −20°C until laboratory analysis.

**Table 1:** Information on the sampling details and characteristics of the field and molecular data. Time/roost sampling points per bat species denote how many times per roost the species was sampled: *M. daubentonii* was sampled from only a single roost (NAU; see Figure 2 for locations of the roost sites in the current study), *E. nilssonii* was sampled separately from two roosting sites (SJÄ, SSA), *M. mystacinus* and *P. auritus* were sampled from the same roost (LAI), and *M. brandtii* was sampled at two locations (RUI), one of which was shared by *P. auritus* (ROT). We found no statistical differences between samples from different bat species in the total reads, total prey species richness, or the average number of prey in each pellet

	All samples	*Myotis daubentonii*	*Eptesicus nilssonii*	*M. brandtii*	*M. mystacinus*	*Plecotus auritus*
Sampling period	29th Apr–7th Aug 2014	30th Apr–7th Aug	15th May–18th Jul	27th May–19th Jul	18th Jul	29th Apr–19th Jul
Pooled samples	51	20	9	10	1	11
Pellets in total	1,215	453	225	250	25	262
Avg. prey species per pellet	3.1 ± 1.4	3.0 ± 1.7	2.9 ± 1.1	3.3 ± 0.9	4.2	3.1 ± 1.6
Total prey reads	5,449,755	1,768,337	1,030,783	1,128,927	119,416	1,402,292
Avg. reads per sample	106,858 ± 52,134	88,417 ± 42,780	114,531 ± 69,513	112,893 ± 50,648	119,416	127,481 ± 51,818
Prey species	547	340	301	329	105	277
Avg. prey species per sample	69.7 ± 23.8	60.6 ± 22.6	71.8 ± 26.9	83.3 ± 23.2	105.0	69.2 ± 17.7

### 2.3 Laboratory work

We aimed to pool 25 droppings (from the same roost and same time point) into each sample to maximize the number of droppings without the need to analyze hundreds of fecal pellets individually. Only four samples included less than 25 droppings, and for these, we pooled every available pellet for the given time point per roost. We focused sampling on roosts inhabited by a single species, and likewise, we intended to pool pellets from a single species into a single pooled sample. In total, we initially sampled 1,252 fecal pellets from the five bat species in this study (Table 1). The DNA was extracted using NucleoSpin® DNA Stool Kit (product nr 740472, Macherey‐Nagel, Düren, Germany) following the manual (version April 2016/Rev. 01) “Protocol for fresh or frozen stool samples” with following modifications: step 1) we used on average 360 mg (±91 mg) of starting material per sample (samples dried only briefly on paper prior to the weighing), and we increased the amount of lysis buffer ST1 to 1,000 µl to increase the amount of supernatant in the subsequent stages; step 2) we used Tissue Lyser II (Cat No. 85300, Qiagen, Hilden, Germany) 2 × 30 s at full speed; step 3) we centrifuged the samples at 13,000 *g* for 5 min, after which the supernatant was transferred into a new tube; and in the final step DNA was eluted into 100 µl of SE buffer.

We used a single primer pair (SFF‐145f: 5′‐GTHACHGCYCAYGCHTTYGTAATAAT‐3′ and SFF‐351r: 5′‐CTCCWGCRTGDGCWAGRTTTCC‐3′; primers and PCR setup from Walker, Williamson, Sanchez, Sobek, & Chambers, 2016) to test the DNA extraction success in the pooled samples and confirm the bat species by molecular analysis and another primer pair to amplify the potential prey (ZBJ‐ArtF1c: 5′‐AGATATTGGAACWTTATATTTTATTTTTGG‐3′ and ZBJ‐ArtR2c: 5′‐WACTAATCAATTWCCAAATCCTCC‐3′; primers and PCR setup from Zeale, Butlin, Barker, Lees, & Jones, 2011). Despite the proposed bias in Zeale primers toward Diptera and Lepidoptera (Clarke, Soubrier, Weyrich, & Cooper, 2014), we chose these for several reasons: (a) These are the most widely applied markers, (b) many species have been detected using exactly the same primers, even though claimed to be nonamplifiable in the earlier criticism, and (c) we wanted to allow comparison of our results with those of other studies using the same primers (Clare et al., 2014; Kaunisto, Roslin, Sääksjärvi, & Vesterinen, 2017; Koskinen et al., 2018; Krüger, Clare, Greif, et al., 2014; Krüger, Clare, Symondson, et al., 2014; Vesterinen et al., 2013, 2016 ; Wirta et al., 2015; Eitzinger et al., 2018). The PCR and library construction closely followed Kaunisto et al. (2017), except we used MyTaq HS Red Mix (product nr BIO‐25048, Bioline, UK) polymerase throughout the protocol. In short, the first‐step PCR reactions included tagged locus‐specific primers targeting either predator or prey COI gene, and the second‐step PCR followed directly after this including Illumina‐specific adapters with a unique dual‐index combination for each single reaction. After this, the individual libraries were pooled (SFF and ZBJ in separate pools at this stage) by equal volume (2 µl each library) and each pool was purified using dual‐SPRI (solid‐phase reversible immobilization) beads as in Vesterinen et al. (2016). To summarize the SPRI method, 80 µl SPRI was added on top of 100 µl library pool, vortexed thoroughly and incubated at room temperature for 5 min. The mix was then briefly centrifuged and placed on a strong magnet until clear, after which the supernatant was removed (shorter than 600 bp fragments in the beads, longer in the supernatant) and 20 µl SPRI was added to the pellet, and then once again vortexed, incubated, centrifuged, and placed on magnet. Supernatant was removed (shorter than 250 bp in the supernatant, longer in the beads), and pellet was washed twice with freshly prepared 70% ethanol and then dried. Then, 100 µl of MQ‐H_2_O was added, vortexed, incubated, centrifuged, and placed on magnet, and subsequently, the purified pool was transferred into a clean Lo‐Bind 1.5 ml Eppendorf tube. We then combined ZBJ (90% of the final pool volume) and SFF (10%) pools into one. See Vesterinen et al. (2016) and Koskinen et al. (2018) for further instructions for how to prepare and use SPRI. The pool included a smaller set of samples (approximately one‐third of the input DNA in the pool) to be used in another study. Sequencing was performed on the Illumina MiSeq platform (Illumina Inc., San Diego, California, USA) by the Turku Centre for Biotechnology, Turku, Finland, using v2 chemistry with 300 cycles and 2 × 150 bp paired‐end read length.

### 2.4 Bioinformatics and prey list construction

The Illumina sequencing yielded 13,219,213 paired‐end reads (SFF: 2,480,440 reads; ZBJ: 10,738,773 reads) identified to samples with unique dual‐index combinations. The reads were uploaded directly from the sequencing facility to CSC servers (IT Center for Science, http://www.csc.fi) for trimming and further analysis. Trimming and quality control of the sequences were conducted according to Kaunisto et al. (2017). Consequently, paired‐end reads were merged (SFF: ~90% reads successfully merged; ZBJ: ~85%) and trimmed for quality using program USEARCH with “fastq_maxee_rate” algorithm with threshold 1 (Edgar, 2010). Primers were removed using python program cutadapt (SFF: ~99% reads passed; ZBJ: ~96%) (Martin, 2011). We then dereplicated reads using USEARCH “fastx_uniques” algorithm with option “minuniquesize 2”, and then, we applied USEARCH UNOISE3 algorithm to cluster these unique reads into ZOTUs (zero‐radius operational taxonomical units; Edgar, 2016). In short, UNOISE algorithm allows the simultaneous a) detection and removal of chimeras (PCR artifacts where two fragments of different origin bind together), point errors (substitutions due to incorrect base calls and gaps due to omitted or spurious base calls), and b) results in ZOTUs (zero‐radius OTUs) that are superior to conventional 97% OTUs for most purposes, because they provide the maximum possible biological resolution given the data available (Edgar, 2016). Finally, reads were mapped back to the original trimmed reads to establish the total number of reads in each sample using USEARCH “otutab” algorithm. After processing, our datasets for this study consisted of 5,449,755 prey reads (produced with primers ZBJ‐ArtF1c and ZBJ‐ArtR2c) and 1,452,602 bat reads (produced with primers SFF‐145f and SFF‐351r). The remaining reads (roughly 30% of total output of the sequencing run; ZBJ: 2,618,342 + SFF: 721,684) were used in another study.

We used the following strict criteria for including prey species in the data: (a) Sequence similarity with the reference sequence had to be at least 98% for the ZOTU to be given any (even higher taxa) assignation, and (b) at least ten reads of the final assigned prey species were required to be present in the final data. We assigned the ZOTUs to species as accurately as possible, utilizing a large reference sequence collection orchestrated by the Finnish Barcode of Life campaign (FinBOL: http://www.finbol.org) and BOLD database (Ratnasingham & Hebert, 2007), and confirmed that all the prey species were actually recorded from (southern) Finland. After the above trimming, we were able to identify and retain 93% of all the prey reads. To account for the even distribution of reads into separate samples, we used ANOVA to test samples from different bat species for differences in the total reads per sample, total prey species richness per sample, and the average number of prey in each pellet (prey richness divided by the number of pooled pellets). The reads originating from bats in the second dataset were used to confirm the bat species identity. The molecular confirmation of bat species revealed a switch in roost occupancy (*M. mystacinus* to *E. nilssonii*) in the middle of the sampling season, which resulted in only one pooled sample of *M. mystacinus*. Also, we removed two mixed samples, containing DNA from two distinct bat species. Labeled raw reads and ZOTUs are available in the Dryad Digital Repository: http://https://doi.org/10.5061/dryad.6880rf1.

A number of metric measurements strongly correlate with the biomass in insects (García‐Barros, 2015; Gruner, 2003). Thus, for data on taxon‐specific prey size (wingspan for Lepidoptera and thorax length for all the other prey taxa) we referred to earlier dietary studies from Finland (Kaunisto et al., 2017; Vesterinen et al., 2016), or to literature or pictures from reference databases. Wingspan for lepidopteran prey was chosen as it was highly available, accessible, and reliable. The prey taxa where the size could not be determined (e.g., due to a compound taxon that was too large to be reliable or informative, such as “Orthoptera sp.”) were omitted from the prey size analysis. For the predator size analysis, we extracted forearm (FA) length measurements from bat banding data collected from the study area. Forearm length is a standard measurement for bats, and it has been shown to highly correlate with the full body length (*R* ^2^ = 0.933; Meng, Zhu, Huang, Irwin, & Zhang, 2016). After discarding repeatedly encountered bat individuals, as well as those with unclear identification or no data on size, we ended up with 1,553 distinct individuals from the bat banding data.

### 2.5 Data analysis

Traditionally, the read count (or read abundance) data produced in metabarcoding studies are directly transformed into presence/absence data, considered to be more cautious and less biased than using read counts. However, the latest opinion on the field seems to suggest that using normalized read abundance data could be even less biased than mere converting to p/a data (Deagle et al., 2018; see also Vesterinen, 2015; Vesterinen et al., 2016). For this reason, we chose to use relative read abundance (RRA: calculated as the proportion of reads per each prey item in each sample). To make the comparison to earlier studies possible, we also prepared the secondary set of analysis using p/a data or more precisely the modified frequency of occurrence (MFO) data throughout the analysis. MFO was calculated as the proportion of occurrences of each prey taxa in each sample scaled to 100% across all prey items (see Deagle et al. (2018) for the terminology and further discussion on the topic).

To begin our data analysis, we calculated prey species accumulation curves to account for sampling adequacy (Colwell & Coddington, 1994). We used R package “iNEXT” to resample the prey reads and frequencies for each bat species and plotted these against accumulated prey species richness (Hsieh, Ma, & Chao, 2016; R Core Team, 2013).

In order to unfold the trophic interactions resolved by the DNA analysis, we used package bipartite (Dormann, Gruber, & Fründ, 2008) implemented in program R to draw interaction webs for each bat predator species using both RRA and MFO data. For those two cases, where two different bat species were observed in the same roost, we constructed additional webs to analyze the diet between separate samples in each location using RRA data. To further estimate patterns among the dietary assemblages of the five species, we used principal coordinates analysis (PCoA) based on Bray–Curtis dissimilarity (Jaccard similarity for presence/absence data) between samples (Davis, 2002; Podani & Miklós, 2002).

Then, to study the effects of predator species and temporal variation (as week number) on variation in prey species composition in each sample, we conducted a permutational multivariate analysis of variance (with Bray–Curtis for RRA and Jaccard for presence/absence data), using 9,999 random permutations to evaluate statistical significance (Anderson, 2001)(PERMANOVA; Anderson, 2001). Analysis of variance was carried out using “adonis” in software R with package “vegan” (Oksanen et al., 2013). Variation was further dissembled using pairwise analysis of variance with package “pairwise.adonis” between all bat species using Bonferroni correction for *p*‐values (Martinez Arbizu, 2017).

Finally, we used information on predator and prey sizes to add dimensions to our attempt to segregate the ecological guilds and predator species. The bat banding data (*n* = 1,553) consisted of unequal sample sizes for the five bat species with unequal variances (Levene's test for homogeneity of variance: *p = 0.0012*), and thus, to compare the forearm lengths (size) of the five bat species, we used a Kruskal–Wallis analysis of variance (nonparametric ANOVA) procedure to compare body size (FA length) as a function of predator size using command “kruskal.test” in R (Kruskal & Wallis, 1952). To further study the difference between bat species pairs, we applied the Tukey and Kramer (Nemenyi) test with Tukey‐Dist approximation for independent samples with R package “PMCMR” (Pohlert, 2014; Sach, 1997, pp. 395–397, 662–664). The same tests were applied to test prey size (wingspan or thorax length as explained above) differences between the bat species.

## 3 RESULTS

### 3.1 General aspects of the diet and the study

**Figure 3:**
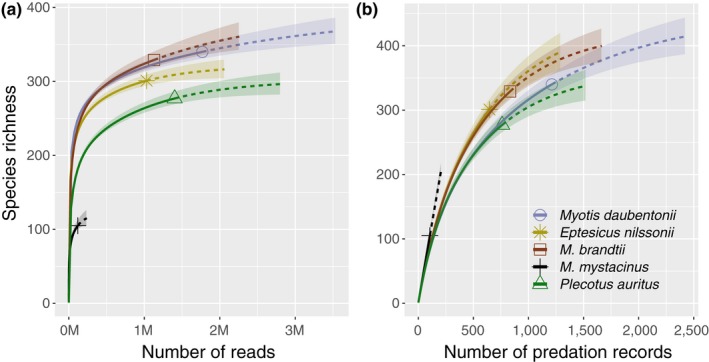
(a) Read‐count‐based and (b) frequency‐of‐occurrence‐based rarefaction (solid line segment) and extrapolation (dotted line segments) sampling curves with 95% confidence intervals (shaded areas) for the five bat's prey species. The solid shapes represent the reference samples

Altogether, we identified 547 distinct prey species in 13 arthropod orders (Table 1). The main prey order for *M. daubentonii* and *E. nilssonii* was Diptera (56% and 77% of all reads, respectively). For *M. brandtii*,* M. mystacinus*, and *P. auritus*, Lepidoptera was the largest prey order (65%, 74%, and 72%, respectively). The only other very abundant prey orders included Trichoptera (15% of reads in *M. daubentonii* diet) and Coleoptera (19% in *P. auritus*). The observed summed prey species richness per bat species varied from 105 prey species to 340 prey species (Tables 1 and 2). From technical point of view, our data show even average distribution of reads across samples (although with high variation), and the average number of prey species per pellet calculated across samples did not differ between bat species (Table 1). The species accumulation curves showed that for *M. mystacinus* the sampling was rather inadequate, but for others more comparable to each other in terms of reads per bat species (Figure 3a), although when using presence/absence data, the curves did not seem to reach the plateau yet (Figure 3b). Nevertheless, we kept *M. mystacinus* in all the analysis, but interpret the results with relevant caution.

**Table 2:** Prey species observed in the current study. For simplicity, prey species are reported as presence or absence for each bat species. First column stands for the prey number used in the *plotweb* analysis (Figures 3 and 4). If species name was not available in the molecular species assignation, the BIN cluster number is reported, as listed in Barcode of Life Database (http://https://v4.boldsystems.org). The bat species are abbreviated as follows: Md = *Myotis daubentonii*, En = *Eptesicus nilssonii*, Mb = *M. brandtii*, Mm = *M. mystacinus*, and Pa = *Plecotus auritus*

No	Prey taxa	*Md*	*En*	*Mb*	*Mm*	*Pa*
	ARACHNIDA					
	**Araneae**					
	Anyphaenidae					
1	*Anyphaena accentuata*	1	1	1	1	1
	Araneidae					
2	*Larinioides patagiatus*	0	1	0	0	0
	Linyphiidae					
3	*Diplostyla concolor*	0	1	0	0	0
4	*Erigone sp*.	0	0	0	0	1
	Philodromidae					
5	*Philodromus cespitum*	0	1	1	0	0
	Theridiidae					
6	*Cryptachaea riparia*	1	0	0	0	0
	Thomisidae					
7	*Xysticus sp. 1*	0	0	1	0	0
8	*Xysticus sp. 2*	1	0	1	0	0
	INSECTA					
	**Blattodea**					
	Ectobiidae					
9	*Ectobius sp*.	0	0	1	1	1
	**Coleoptera**					
	Cantharidae					
10	*Podabrus alpinus*	0	1	0	0	0
	Carabidae					
11	*Acupalpus parvulus*	0	1	0	0	1
12	*Badister dilatatus*	0	1	0	0	0
13	*Pterostichus adstrictus*	1	1	1	0	1
14	*Pterostichus melanarius*	1	1	1	1	1
15	*Pterostichus nigrita*	1	0	0	0	0
	Cerambycidae					
16	*Acanthocinus aedilis*	0	1	0	0	1
17	*Coleoptera sp*.	0	1	0	0	0
	Curculionidae					
18	*Brachyderes incanus*	0	0	0	0	1
19	*Strophosoma capitatum*	0	0	0	0	1
	Dytiscidae					
20	*Laccophilus comes*	0	0	1	0	0
	Gyrinidae					
21	*Orectochilus villosus*	1	1	1	0	1
	Melyridae					
22	*Dasytes plumbeus*	1	0	1	0	0
	Oedemeridae					
23	*Calopus serraticornis*	0	1	1	0	1
	Staphylinidae					
24	*Dropephylla ioptera*	0	1	0	0	0
25	*Nudobius lentus*	0	0	0	0	1
	**Diptera**					
	Anisopodidae					
26	*Sylvicola cinctus*	1	1	1	0	0
27	*Sylvicola fenestralis*	0	1	1	1	1
	Anthomyiidae					
28	*Delia florilega*	0	1	0	0	0
29	*Delia platura*	1	1	0	0	1
30	*Pegomya rubivora*	1	0	0	0	1
31	*Pegomya sp*.	0	1	0	0	0
32	*Pegoplata annulata*	1	1	0	0	1
33	*Zaphne ambigua*	0	1	0	0	0
	Anthomyzidae					
34	*Anthomyza sp*.	1	0	1	0	1
	Calliphoridae					
35	*Melinda viridicyanea*	1	0	0	0	0
	Cecidomyiidae					
36	*CecidInt35 sp. BOLD:ACB9926*	0	0	1	0	0
37	*Cecidomyiidae sp*.	1	0	1	0	0
	Ceratopogonidae					
38	*Palpomyia lineata*	1	1	1	0	0
	Chaoboridae					
39	*Chaoborus flavicans*	1	1	0	0	0
40	*Chaoborus sp. BOLD:AAG5462*	1	1	1	0	0
	Chironomidae					
41	*Ablabesmyia aspera*	1	0	1	0	0
42	*Ablabesmyia monilis*	1	0	0	0	0
43	*Arctopelopia barbitarsis*	1	1	1	0	1
44	*Chironomidae sp. BOLD:ACP1316*	1	0	0	0	0
45	*Chironomidae sp. BOLD:ACQ8800*	1	1	1	0	1
46	*Chironomidae sp. BOLD:ACU9532*	1	0	0	0	1
47	*Chironominae sp*.	1	1	0	0	0
48	*Chironomus sp. BOLD:AAI4299*	1	1	1	0	0
49	*Chironomus sp. BOLD:AAI4301*	1	1	1	0	0
50	*Chironomus sp.1*	1	1	1	0	1
51	*Chironomus sp.2*	1	1	1	0	1
52	*Cladopelma sp*.	1	1	0	0	0
53	*Cladopelma sp. 1TE*	1	1	0	1	0
54	*Conchapelopia melanops*	1	1	1	0	0
55	*Conchapelopia sp. BOLD:ACQ3496*	1	0	0	0	0
56	*Cricotopus bicinctus*	1	1	1	0	1
57	*Cricotopus sp*.	1	1	1	0	1
58	*Cricotopus sylvestris*	1	0	1	0	0
59	*Cricotopus triannulatus*	1	0	0	0	0
60	*Cryptochironomus supplicans*	1	1	0	0	0
61	*Demicryptochironomus sp*.	0	1	0	0	0
62	*Dicrotendipes lobiger*	0	1	1	0	0
63	*Dicrotendipes nervosus*	1	1	1	0	0
64	*Dicrotendipes tritomus*	0	1	0	0	0
65	*Endochironomus tendens*	1	1	0	0	0
66	*Glyptotendipes barbipes*	0	1	0	0	0
67	*Glyptotendipes cauliginellus*	1	1	1	0	1
68	*Glyptotendipes lobiferus*	1	1	1	0	1
69	*Glyptotendipes sp*.	1	0	0	0	0
70	*Glyptotendipes sp. BOLD:ACG4324*	1	1	0	0	0
71	*Heterotrissocladius marcidus*	1	0	0	0	1
72	*Kiefferulus sp*.	1	0	1	0	0
73	*Metriocnemus sp. 3ES*	0	0	1	0	0
74	*Microchironomus tener*	0	0	1	0	0
75	*Microtendipes chloris*	1	1	1	0	1
76	*Microtendipes pedellus*	1	1	1	0	1
77	*Microtendipes sp*.	1	1	1	0	1
78	*Orthocladiinae sp*.	1	1	1	0	1
79	*Parachironomus digitalis*	1	0	1	0	1
80	*Parachironomus monochromus*	1	0	0	0	0
81	*Paracladopelma sp.1*	1	0	0	0	1
82	*Paracladopelma sp.2*	1	0	0	0	0
83	*Paratanytarsus dissimilis*	0	0	1	0	0
84	*Polypedilum convictum*	1	0	0	0	0
85	*Polypedilum nubeculosum*	1	1	1	0	1
86	*Polypedilum pedestre*	0	0	1	0	0
87	*Polypedilum sordens*	1	1	1	0	0
88	*Polypedilum sp*.	1	0	1	0	0
89	*Polypedilum sp. BOLD:ACR0701*	1	0	0	0	0
90	*Procladius culiciformis*	1	1	1	0	0
91	*Procladius nigriventris*	1	1	0	0	0
92	*Procladius sp. 1ES*	1	1	1	0	1
93	*Procladius sp. BOLD:AAG5487*	1	1	1	0	1
94	*Psectrocladius limbatellus*	0	1	0	0	0
95	*Psectrocladius octomaculatus*	0	1	0	0	0
96	*Psectrocladius sp*.	1	0	0	0	0
97	*Psectrotanypus varius*	1	0	0	0	0
98	*Stictochironomus sp. 3TE*	1	1	1	0	1
99	*Tanytarsus eminulus*	1	0	1	0	0
100	*Tanytarsus mendax*	1	1	1	0	0
101	*Thienemannimyia carnea*	1	1	1	1	1
102	*Xenochironomus xenolabis*	1	1	0	0	1
103	*Zavrelimyia sp*.	1	0	1	0	0
	Chloropidae					
104	*Thaumatomyia notata*	0	1	0	0	0
105	*Thaumatomyia sp*.	0	1	0	0	1
106	*Thaumatomyia sp. BOLD:ACX2752*	0	1	0	0	0
	Culicidae					
107	*Aedes cinereus*	1	1	1	0	0
108	*Aedes vexans*	0	1	0	0	1
109	*Anopheles claviger*	0	0	1	0	0
110	*Anopheles messeae*	1	1	1	1	0
111	*Culex pipiens*	1	1	1	1	1
112	*Culicidae sp. 1*	0	1	0	0	0
113	*Culicidae sp. 2*	0	1	0	0	0
114	*Culiseta annulata*	0	1	1	0	0
115	*Culiseta morsitans*	0	1	1	0	1
116	*Culiseta ochroptera*	0	1	0	0	1
117	*Ochlerotatus cataphylla*	0	0	1	0	0
118	*Ochlerotatus communis*	1	1	1	0	1
119	*Ochlerotatus excrucians*	0	1	0	0	0
120	*Ochlerotatus punctor*	0	1	1	0	0
	Dolichopodidae					
121	*Gymnopternus sp*.	0	0	1	1	0
	Drosophilidae					
122	*Drosophilidae sp*.	0	0	1	0	1
123	*Scaptomyza pallida*	0	1	0	0	0
	Empididae					
124	*Rhamphomyia anaxo*	1	0	1	0	0
125	*Rhamphomyia caesia*	0	0	1	0	0
126	*Rhamphomyia nigripennis*	1	1	1	0	1
127	*Rhamphomyia nr. anaxo*	1	0	1	0	0
128	*Rhamphomyia sp*.	0	1	0	0	0
129	*Rhamphomyia umbripennis*	0	0	1	0	0
130	*Rhamphomyia valga*	0	1	1	0	0
	Fanniidae					
131	*Fannia minutipalpis*	0	0	1	0	0
132	*Fannia sociella*	1	1	0	0	0
	Heleomyzidae					
133	*Suillia bicolor*	0	0	0	0	1
	Hippoboscidae					
134	*Nycteribia kolenati*	1	1	1	0	1
	Hybotidae					
135	*Bicellaria simplicipes*	0	1	0	0	1
	Keroplatidae					
136	*Macrocera stigma*	0	0	1	0	0
	Limoniidae					
137	*Austrolimnophila unica*	0	1	1	0	1
138	*Dicranomyia didyma*	1	0	0	0	0
139	*Dicranomyia frontalis*	0	0	0	1	0
140	*Dicranomyia modesta*	1	1	1	0	0
141	*Dicranomyia sp*.	1	1	0	1	0
142	*Eloeophila maculata*	1	0	1	1	0
143	*Erioptera divisa*	1	0	1	0	0
144	*Erioptera sp*.	1	1	1	0	0
145	*Gonomyia tenella*	0	1	0	0	0
146	*Helius longirostris*	1	1	1	0	1
147	*Limonia nubeculosa*	1	0	0	0	0
148	*Limonia trivittata*	1	0	1	1	0
149	*Metalimnobia bifasciata*	1	0	0	1	1
150	*Metalimnobia quadrinotata*	1	1	1	1	1
151	*Molophilus sp*.	0	0	0	0	1
152	*Phylidorea squalens*	0	1	0	0	0
153	*Rhipidia maculata*	1	1	1	0	1
154	*Symplecta stictica*	1	0	1	0	0
	Muscidae					
155	*Helina evecta*	1	1	1	0	0
156	*Hydrotaea armipes*	0	0	0	1	0
157	*Hydrotaea irritans*	0	0	0	1	1
158	*Muscina levida*	0	0	0	0	1
159	*Mydaea new sp. nr urbana*	0	1	0	0	0
160	*Polietes lardarius*	1	0	1	0	1
161	*Thricops diaphanus*	0	0	0	0	1
162	*Thricops rufisquamus*	0	1	1	0	0
	Mycetophilidae					
163	*Exechia seriata*	0	1	0	0	0
164	*Phronia sp*.	0	0	1	1	0
165	*Sciophila lutea*	1	1	1	0	0
166	*Sciophila pseudoflexuosa*	0	0	1	0	0
	Pediciidae					
167	*Pedicia rivosa*	0	1	0	0	1
168	*Pediciidae sp*.	1	1	1	0	1
169	*Ula mixta*	1	1	1	0	1
170	*Ula sylvatica*	1	1	1	0	1
	Psychodidae					
171	*Psychoda lobata*	1	1	1	0	1
172	*Psychoda sp*.	1	1	1	1	1
173	*Telmatoscopus advena*	1	0	1	0	0
	Rhagionidae					
174	*Rhagio scolopaceus*	1	1	1	0	1
	Rhinophoridae					
175	*Paykullia maculata*	1	0	1	0	0
	Scathophagidae					
176	*Scathophaga suilla*	0	1	1	0	0
	Sciaridae					
177	*Sciaridae sp*.	1	1	1	0	0
	Simuliidae					
178	*Simulium equinum*	1	0	0	0	1
179	*Simulium noelleri*	1	1	1	0	1
180	*Simulium ornatum*	1	0	1	0	0
181	*Simulium vernum*	0	1	1	0	1
	Stratiomyidae					
182	*Beris chalybata*	0	0	1	0	0
	Syrphidae					
183	*Meliscaeva cinctella*	0	0	0	0	1
184	*Syrphus torvus*	0	1	1	0	0
185	*Syrphus vitripennis*	1	1	1	0	1
186	*Xanthandrus comtus*	0	1	1	0	0
	Tachinidae					
187	*Bactromyia aurulenta*	1	1	1	0	1
188	*Ceromya silacea*	0	0	1	1	1
189	*Cyzenis albicans*	1	0	0	0	0
190	*Eloceria delecta*	0	0	0	0	1
191	*Loewia foeda*	1	1	1	0	1
192	*Macquartia dispar*	1	0	0	0	0
193	*Oswaldia muscaria*	0	0	1	0	1
194	*Pales pavida*	0	0	0	0	1
195	*Phorocera obscura*	1	1	1	0	0
196	*Siphona geniculata*	0	0	1	0	0
	Tipulidae					
197	*Nephrotoma aculeata*	1	1	1	0	1
198	*Nephrotoma lunulicornis*	1	1	0	0	0
199	*Tipula fascipennis*	1	1	1	1	1
200	*Tipula fulvipennis*	0	1	1	0	0
201	*Tipula lateralis*	1	0	0	0	0
202	*Tipula lunata*	0	1	1	1	1
203	*Tipula maxima*	1	1	0	0	1
204	*Tipula nubeculosa*	0	1	0	0	1
205	*Tipula paludosa*	1	1	0	0	1
206	*Tipula pierrei*	1	1	1	0	1
207	*Tipula scripta*	1	1	1	1	1
208	*Tipula sp. BOLD:AAF9041*	1	1	0	0	0
209	*Tipula truncorum*	1	1	1	1	1
210	*Tipulidae sp*.	0	1	0	0	1
	Trichoceridae					
211	*Trichocera regelationis*	1	1	1	0	1
212	*Trichocera sp*.	1	0	1	0	0
	**Ephemeroptera**					
	Baetidae					
213	*Procloeon bifidum*	1	0	0	0	0
	Caenidae					
214	*Caenis horaria*	1	1	1	0	1
	Ephemeridae					
215	*Ephemera vulgata*	1	1	0	0	0
	Heptageniidae					
216	*Heptagenia sulphurea*	1	1	1	0	1
	Siphlonuridae					
217	*Siphlonurus alternatus*	1	0	1	0	0
	**Hemiptera**					
	Aphididae					
218	*Euceraphis betulae*	0	1	1	0	1
219	*Euceraphis punctipennis*	0	1	0	0	1
	Cicadellidae					
220	*Fagocyba douglasi*	0	0	1	0	0
	Miridae					
221	*Lygus pratensis*	0	1	1	0	1
222	*Neolygus contaminatus*	1	0	1	1	0
	**Hymenoptera**					
	Braconidae					
223	*Choeras jft30*	0	1	1	0	0
224	*Hymenoptera sp*.	1	0	1	0	0
	Ichneumonidae					
225	*Astiphromma splenium*	0	0	1	0	1
226	*Diadegma majale*	0	0	1	0	0
227	*Hyposoter PRO‐3*	0	0	1	0	0
228	*Mesochorus sp*.	1	0	0	0	0
229	*Mesochorus vitticollis*	0	1	1	0	1
230	*Pleolophus sp*.	0	0	0	0	1
	Tenthredinidae					
231	*Dolerus vestigialis*	1	0	0	0	1
232	*Pachyprotasis rapae*	1	1	0	0	0
	**Lepidoptera**					
	Adelidae					
233	*Nematopogon swammerdamellus*	1	1	1	0	0
	Arctiidae					
234	*Atolmis rubricollis*	1	0	1	0	1
235	*Eilema depressum*	0	0	0	1	0
	Argyresthiidae					
236	*Argyresthia abdominalis*	1	0	0	0	0
237	*Argyresthia bergiella*	1	1	1	1	1
238	*Argyresthia goedartella*	1	1	1	1	1
239	*Argyresthia retinella*	0	1	1	0	1
	Batrachedridae					
240	*Batrachedra pinicolella*	1	0	1	0	1
	Bucculatricidae					
241	*Bucculatrix cidarella*	0	0	1	0	0
242	*Bucculatrix thoracella*	1	0	1	0	0
243	*Bucculatrix ulmella*	1	1	1	0	1
	Coleophoridae					
244	*Coleophora betulella*	1	1	1	0	1
245	*Coleophora kuehnella*	0	1	1	0	0
246	*Coleophora spinella*	1	1	1	1	1
247	*Coleophora versurella*	1	1	0	1	1
	Cosmopterigidae					
248	*Limnaecia phragmitella*	1	0	0	0	0
249	*Sorhagenia janiszewskae*	1	0	0	0	0
	Crambidae					
250	*Acentria ephemerella*	1	0	0	0	1
251	*Agriphila inquinatella*	1	0	0	0	1
252	*Agriphila selasella*	1	1	0	0	1
253	*Agriphila straminella*	1	0	0	0	0
254	*Calamotropha paludella*	1	1	0	0	0
255	*Chrysoteuchia culmella*	0	1	1	0	1
256	*Crambus lathoniellus*	1	0	0	0	0
257	*Crambus pascuellus*	0	0	0	1	1
258	*Donacaula mucronella*	1	1	1	0	1
259	*Elophila nymphaeata*	1	0	0	1	1
260	*Evergestis extimalis*	1	0	1	0	1
261	*Nymphula nitidulata*	1	0	1	0	0
262	*Ostrinia nubilalis*	1	0	1	0	0
263	*Scoparia ancipitella*	1	0	1	1	1
264	*Scoparia subfusca*	1	0	0	0	0
265	*Udea lutealis*	1	0	0	1	0
	Depressariidae					
266	*Agonopterix angelicella*	1	1	1	0	1
267	*Agonopterix arenella*	1	0	1	0	1
268	*Agonopterix ciliella*	1	0	1	1	1
269	*Agonopterix heracliana*	1	1	1	0	1
270	*Agonopterix propinquella*	1	0	1	0	0
271	*Depressaria daucella*	1	1	1	0	1
272	*Depressaria emeritella*	1	1	1	0	1
273	*Depressaria libanotidella*	1	1	1	0	1
274	*Depressaria olerella*	1	1	1	0	1
275	*Depressaria radiella*	1	0	0	0	0
276	*Depressaria sordidatella*	1	1	0	0	1
	Drepanidae					
277	*Drepana falcataria*	1	0	0	0	0
278	*Falcaria lacertinaria*	1	0	1	0	0
279	*Tethea or*	0	0	0	0	1
280	*Tetheella fluctuosa*	1	1	1	0	1
	Elachistidae					
281	*Elachista adscitella*	0	0	1	1	1
	Endromidae					
282	*Endromis versicolora*	0	1	1	0	1
	Epermeniidae					
283	*Epermenia illigerella*	1	0	0	0	0
	Erebidae					
284	*Calliteara pudibunda*	0	1	1	0	1
285	*Diacrisia sannio*	1	1	0	0	1
286	*Herminia tarsipennalis*	0	0	1	0	1
287	*Hypena crassalis*	0	1	0	0	1
288	*Macrochilo cribrumalis*	1	1	1	1	1
289	*Rivula sericealis*	0	0	1	1	0
290	*Scoliopteryx libatrix*	0	0	0	0	1
291	*Spilarctia luteum*	1	0	0	0	1
	Gelechiidae					
292	*Carpatolechia fugitivella*	0	0	1	0	0
293	*Carpatolechia proximella*	1	1	1	0	1
294	*Caryocolum vicinella*	1	1	1	1	1
295	*Chionodes electella*	1	1	1	1	1
296	*Chionodes lugubrella*	1	1	1	0	1
297	*Dichomeris alacella*	0	0	1	0	0
298	*Exoteleia dodecella*	1	1	1	1	1
299	*Gelechia muscosella*	1	0	0	0	0
300	*Gelechia nigra*	1	1	0	1	0
301	*Gelechia sororculella*	1	0	0	0	1
302	*Helcystogramma rufescens*	1	1	0	0	1
303	*Monochroa lutulentella*	1	1	1	0	1
304	*Neofriseria peliella*	1	1	1	1	0
305	*Psoricoptera gibbosella*	1	1	1	0	1
306	*Recurvaria leucatella*	1	0	1	0	0
307	*Scrobipalpa atriplicella*	1	0	1	0	0
308	*Teleiopsis diffinis*	0	0	1	0	0
	Geometridae					
309	*Aethalura punctulata*	1	0	0	0	1
310	*Agriopis aurantiaria*	1	1	1	1	1
311	*Alcis repandata*	0	0	1	0	1
312	*Bupalus piniaria*	0	1	0	1	1
313	*Cabera pusaria*	0	0	1	0	1
314	*Cleora cinctaria*	0	0	1	1	1
315	*Crocallis elinguaria*	1	1	1	0	1
316	*Deileptenia ribeata*	1	1	1	0	1
317	*Ectropis crepuscularia*	1	1	0	0	1
318	*Epirrhoe alternata*	0	1	0	0	0
319	*Epirrita autumnata*	0	0	1	0	1
320	*Eupithecia abietaria*	0	1	0	0	1
321	*Eupithecia indigata*	0	1	0	0	1
322	*Eupithecia lanceata*	1	1	1	1	1
323	*Eupithecia plumbeolata*	0	1	1	1	1
324	*Eupithecia subfuscata*	1	0	0	0	1
325	*Eupithecia tantillaria*	0	1	0	0	1
326	*Eupithecia tenuiata*	1	0	0	0	1
327	*Eupithecia virgaureata*	1	0	0	0	0
328	*Gandaritis pyraliata*	1	0	0	0	0
329	*Geometridae sp*.	1	1	1	0	1
330	*Idaea dimidiata*	1	0	0	1	1
331	*Idaea emarginata*	1	1	1	0	0
332	*Lomaspilis marginata*	0	1	0	0	0
333	*Macaria liturata*	1	1	1	1	1
334	*Odontopera bidentata*	1	0	0	0	1
335	*Paradarisa consonaria*	0	1	0	0	1
336	*Pasiphila rectangulata*	0	0	1	0	0
337	*Plagodis pulveraria*	0	1	0	0	1
338	*Rheumaptera undulata*	0	0	0	0	1
339	*Scopula floslactata*	1	0	0	0	1
340	*Scopula immutata*	1	0	0	0	0
341	*Selenia dentaria*	1	0	1	0	1
342	*Xanthorhoe montanata*	1	1	1	0	0
343	*Xanthorhoe quadrifasciata*	1	0	1	1	1
344	*Xanthorhoe spadicearia*	0	1	0	0	0
	Glyphipterigidae					
345	*Orthotelia sparganella*	1	0	1	0	0
	Gracillariidae					
346	*Caloptilia alchimiella*	0	0	1	0	0
347	*Caloptilia betulicola*	0	1	1	0	0
348	*Caloptilia elongella*	0	1	1	0	0
349	*Caloptilia hemidactylella*	1	0	1	0	0
350	*Caloptilia populetorum*	0	1	1	0	0
351	*Parornix betulae*	1	1	1	0	0
352	*Parornix devoniella*	1	1	1	0	1
353	*Phyllonorycter harrisella*	0	0	1	0	0
	Hepialidae					
354	*Pharmacis fusconebulosa*	0	0	1	1	1
	Lasiocampidae					
355	*Dendrolimus pini*	1	1	1	0	1
356	*Lasiocampa quercus*	1	1	1	0	1
357	*Macrothylacia rubi*	1	1	1	0	1
	Lyonetiidae					
358	*Lyonetia clerkella*	0	1	1	0	0
	Lypusidae					
359	*Pseudatemelia elsae*	0	1	0	0	0
360	*Pseudatemelia josephinae*	1	1	0	1	1
	Momphidae					
361	*Mompha sturnipennella*	1	0	1	0	0
362	*Mompha subbistrigella*	1	1	1	0	0
	Noctuidae					
363	*Acronicta auricoma*	1	0	0	0	0
364	*Acronicta rumicis*	1	0	0	0	1
365	*Agrochola helvola*	0	0	0	0	1
366	*Agrotis clavis*	1	1	1	1	1
367	*Agrotis exclamationis*	1	1	1	0	1
368	*Allophyes oxyacanthae*	0	0	1	0	1
369	*Apamea crenata*	0	0	1	0	1
370	*Apamea remissa*	1	1	1	1	1
371	*Apamea scolopacina*	0	0	0	0	1
372	*Apamea sordens*	1	1	1	0	1
373	*Autographa gamma*	1	1	1	0	1
374	*Autographa pulchrina*	0	0	0	0	1
375	*Brachionycha nubeculosa*	1	0	0	0	1
376	*Caradrina morpheus*	1	1	1	1	1
377	*Cerastis rubricosa*	1	1	1	1	1
378	*Charanyca ferruginea*	1	1	1	1	1
379	*Chloantha hyperici*	0	0	1	0	0
380	*Colocasia coryli*	0	1	0	0	1
381	*Conistra rubiginea*	1	1	1	1	1
382	*Conistra vaccinii*	1	1	1	1	1
383	*Diarsia rubi*	1	0	0	0	1
384	*Eurois occultus*	1	1	1	1	1
385	*Hada plebeja*	1	0	1	1	1
386	*Helotropha leucostigma*	1	1	1	1	1
387	*Hoplodrina octogenaria*	1	1	1	1	1
388	*Hydraecia micacea*	1	0	1	0	1
389	*Hyppa rectilinea*	1	0	0	0	1
390	*Lenisa geminipuncta*	0	1	0	0	1
391	*Lithophane furcifera*	0	0	0	0	1
392	*Lithophane socia*	1	1	0	1	1
393	*Mesapamea secalis*	0	1	1	1	1
394	*Mniotype bathensis*	0	1	0	0	1
395	*Oligia latruncula*	0	0	0	1	1
396	*Orthosia gothica*	1	1	1	1	1
397	*Orthosia opima*	1	1	1	1	1
398	*Panolis flammea*	1	1	1	1	1
399	*Panthea coenobita*	1	1	0	0	1
400	*Polia hepatica*	1	0	1	0	1
401	*Protolampra sobrina*	0	0	0	0	1
402	*Subacronicta megacephala*	0	1	0	0	1
403	*Trachea atriplicis*	0	0	0	0	1
404	*Xestia triangulum*	1	0	1	1	1
405	*Xylena vetusta*	1	1	1	1	1
	Nolidae					
406	*Nycteola degenerana*	0	1	1	1	1
407	*Nycteola revayana*	1	0	1	0	1
	Notodontidae					
408	*Cerura vinula*	0	0	0	0	1
409	*Notodonta dromedarius*	1	1	0	0	1
410	*Ptilodon capucinus*	1	0	0	0	0
	Nymphalidae					
411	*Argynnis paphia*	0	1	1	0	1
	Oecophoridae					
412	*Crassa tinctella*	1	0	1	1	0
413	*Denisia obscurella*	1	0	1	0	1
414	*Denisia stipella*	0	1	1	0	0
	Pieridae					
415	*Colias palaeno*	0	1	0	0	0
	Plutellidae					
416	*Plutella xylostella*	1	1	1	0	1
	Praydidae					
417	*Prays fraxinella*	0	0	1	0	0
	Psychidae					
418	*Taleporia tubulosa*	0	1	0	0	0
	Pterophoridae					
419	*Gillmeria pallidactyla*	1	0	1	1	0
	Pyralidae					
420	*Dioryctria abietella*	0	0	0	1	1
	Saturniidae					
421	*Aglia tau*	0	1	0	0	1
422	*Saturnia pavonia*	0	0	1	0	1
	Sphingidae					
423	*Deilephila elpenor*	0	0	0	0	1
	Tineidae					
424	*Morophaga choragella*	0	0	1	0	0
425	*Nemapogon nigralbella*	0	0	1	0	0
426	*Nemaxera betulinella*	0	0	1	0	0
427	*Niditinea striolella*	0	0	1	0	0
428	*Triaxomera fulvimitrella*	1	0	1	0	0
	Tischeriidae					
429	*Tischeria ekebladella*	0	1	1	0	0
	Tortricidae					
430	*Acleris forsskaleana*	1	0	1	1	1
431	*Acleris lipsiana*	1	0	1	1	1
432	*Acleris logiana*	1	1	1	0	1
433	*Acleris notana*	1	0	1	0	1
434	*Adoxophyes orana*	1	1	1	1	1
435	*Aethes smeathmanniana*	1	1	1	0	1
436	*Agapeta hamana*	0	1	0	0	0
437	*Aleimma loeflingiana*	0	1	1	1	1
438	*Ancylis badiana*	1	0	0	0	0
439	*Ancylis laetana*	0	0	1	0	0
440	*Ancylis mitterbacheriana*	1	0	1	0	0
441	*Ancylis myrtillana*	1	1	0	0	1
442	*Aphelia paleana*	0	1	0	0	0
443	*Apotomis fraterculana*	1	1	0	1	0
444	*Apotomis infida*	1	0	0	0	0
445	*Archips podanus*	1	0	1	0	1
446	*Bactra lancealana*	1	0	0	0	0
447	*Celypha rivulana*	1	0	0	0	0
448	*Clepsis spectrana*	1	0	0	0	0
449	*Cnephasia asseclana*	0	0	1	0	0
450	*Cnephasia stephensiana*	1	1	1	1	1
451	*Cochylis nana*	1	1	1	0	0
452	*Eana argentana*	1	0	1	0	0
453	*Eana incanana*	1	1	1	1	1
454	*Enarmonia formosana*	0	0	1	0	0
455	*Epiblema scutulana*	1	0	1	0	1
456	*Epinotia bilunana*	0	1	1	0	0
457	*Epinotia cinereana*	0	0	0	1	0
458	*Epinotia nisella*	1	0	1	1	0
459	*Epinotia signatana*	1	0	1	0	0
460	*Epinotia solandriana*	1	0	0	0	0
461	*Epinotia tedella*	0	0	1	1	0
462	*Epinotia tenerana*	0	1	1	1	0
463	*Epinotia tetraquetrana*	0	1	1	0	0
464	*Eucosma cana*	1	0	1	0	1
465	*Eucosma hohenwartiana*	1	0	1	0	0
466	*Eudemis porphyrana*	1	0	1	0	0
467	*Gypsonoma dealbana*	0	1	1	1	0
468	*Hedya nubiferana*	1	1	1	1	0
469	*Hedya ochroleucana*	1	0	0	0	0
470	*Lobesia reliquana*	0	0	1	0	0
471	*Metendothenia atropunctana*	0	1	1	1	0
472	*Orthotaenia undulana*	1	1	1	1	1
473	*Pandemis cerasana*	0	1	1	0	0
474	*Pandemis cinnamomeana*	0	1	1	1	0
475	*Paramesia gnomana*	1	0	1	0	1
476	*Phalonidia udana*	0	0	1	0	0
477	*Piniphila bifasciana*	0	1	0	0	0
478	*Ptycholoma lecheana*	0	0	1	0	1
479	*Rhopobota naevana*	1	1	1	1	1
480	*Rhyacionia buoliana*	0	1	1	0	1
481	*Syndemis musculana*	0	1	1	0	0
482	*Thiodia citrana*	1	1	1	0	1
483	*Tortrix viridana*	1	1	1	1	1
484	*Zeiraphera isertana*	0	0	1	0	0
485	*Zeiraphera ratzeburgiana*	1	0	0	1	0
	Yponomeutidae					
486	*Argyresthia arceuthina*	1	0	1	0	0
487	*Argyresthia brockeella*	0	0	0	1	0
488	*Argyresthia conjugella*	0	1	1	0	1
489	*Argyresthia glabratella*	1	1	1	0	1
490	*Cedestis gysseleniella*	1	1	1	1	1
491	*Paraswammerdamia conspersella*	1	0	1	0	1
492	*Paraswammerdamia nebulella*	1	0	1	0	1
	Ypsolophidae					
493	*Ypsolopha asperella*	0	1	0	0	0
494	*Ypsolopha falcella*	1	1	1	0	0
495	*Ypsolopha parenthesella*	1	1	1	0	1
496	*Ypsolopha scabrella*	1	0	1	0	1
497	*Ypsolopha sylvella*	1	0	1	0	0
498	*Ypsolopha ustella*	1	0	1	0	1
	**Megaloptera**					
	Sialidae					
499	*Sialis lutaria*	1	0	0	0	0
	**Neuroptera**					
	Chrysopidae					
500	*Chrysopa pallens*	1	1	1	0	1
501	*Chrysoperla carnea*	1	1	0	0	1
502	*Cunctochrysa albolineata*	0	0	0	1	0
	Hemerobiidae					
503	*Hemerobius contumax*	1	1	1	1	1
504	*Hemerobius fenestratus*	0	1	1	1	1
505	*Hemerobius humulinus*	1	1	1	1	1
506	*Hemerobius pini*	0	1	0	1	1
507	*Hemerobius stigma*	1	1	1	1	1
508	*Wesmaelius concinnus*	1	1	1	1	1
509	Neuroptera sp.	0	1	1	0	0
	Sisyridae					
510	*Sisyra nigra*	1	0	0	0	1
	**Orthoptera**					
511	*Orthoptera sp*.	0	0	0	0	1
	**Psocodea**					
	Peripsocidae					
512	*Peripsocus subfasciatus*	1	0	1	0	1
	**Trichoptera**					
	Goeridae					
513	*Goera pilosa*	1	1	1	0	1
	Lepidostomatidae					
514	*Lepidostoma hirtum*	1	1	1	0	0
	Leptoceridae					
515	*Athripsodes cinereus*	1	1	0	0	0
516	*Ceraclea albimacula*	1	0	1	0	0
517	*Ceraclea annulicornis*	1	0	0	0	0
518	*Ceraclea dissimilis*	1	0	0	0	0
519	*Ceraclea excisa*	1	0	0	0	0
520	*Ceraclea fulva*	1	1	0	1	0
521	*Ceraclea senilis*	0	1	0	0	0
522	*Mystacides azureus*	1	1	0	0	0
523	*Mystacides longicornis*	0	1	0	0	0
524	*Mystacides nigra*	1	1	0	0	0
525	*Oecetis furva*	1	1	0	0	0
526	*Oecetis lacustris*	1	1	1	0	0
527	*Oecetis ochracea*	0	1	0	0	0
528	*Oecetis testacea*	1	1	0	0	0
529	*Triaenodes detruncatus*	1	0	0	0	0
	Limnephilidae					
530	*Glyphotaelius pellucidus*	1	1	0	0	1
531	*Limnephilus affinis*	1	1	1	0	1
532	*Limnephilus flavicornis*	0	1	0	0	0
533	*Limnephilus fuscicornis*	1	1	0	0	1
534	*Micropterna sequax*	0	0	1	0	1
535	*Rhadicoleptus alpestris*	0	1	1	0	1
536	*Stenophylax lateralis*	0	0	0	0	1
	Molannidae					
537	*Molanna angustata*	1	0	0	0	0
	Phryganeidae					
538	*Agrypnia obsoleta*	1	0	1	0	0
539	*Agrypnia pagetana*	0	1	0	0	1
540	*Agrypnia varia*	0	1	1	0	0
541	*Phryganea grandis*	1	1	1	0	0
	Polycentropodidae					
542	*Cyrnus trimaculatus*	1	0	0	0	0
543	*Plectrocnemia conspersa*	1	1	1	0	0
544	*Polycentropus flavomaculatus*	1	0	1	0	0
	Psychomyiidae					
545	*Lype phaeopa*	1	0	0	0	0
546	*Psychomyia pusilla*	1	1	0	0	0
	Rhyacophilidae					
547	*Rhyacophila nubila*	1	0	0	0	0

### 3.2 Dietary patterns of the studied bats

**Figure 4:**
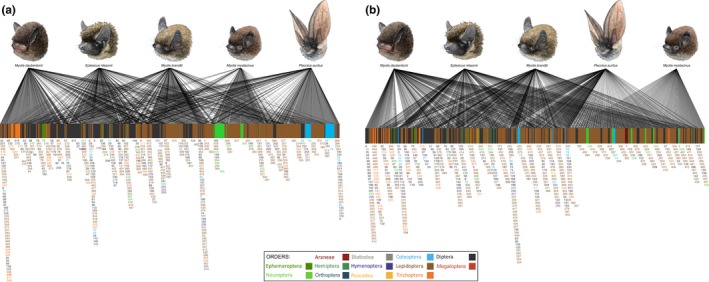
Food webs of the bat predator species and their prey species visualizing the differences in the diet. The pictures in the upper row represent predators in each web and the blocks in the lower row the prey species. A line connecting a predator with a prey represents a detected predation record, and the thickness of the line represents (a) the relative read abundance (RRA) or (b) modified proportional frequency (MFO) of each predation record. See the “Data analysis” in the main text for details on the RRA and MFO. The numbers below the lower blocks correspond to the prey numbers in the Table 2

**Figure 5:**
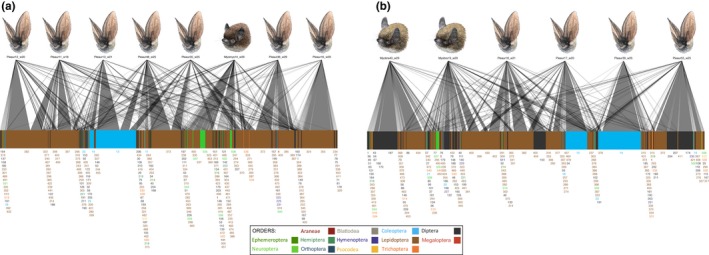
Food webs in the two roosting sites where two different bat species were sampled to show that the bat species consumed dissimilar prey, even when collected on the same site during same time. (a) Laiterla roost food web shows that *M. mystacinus* is fond of soft‐bodied insects, such as Neuroptera, whereas *P. auritus* diet consists of larger carabid beetles. (b) Rotholma roost, where the two *M. brandtii* sample contains different Diptera and Hymenoptera prey, compared to the *P. auritus*. The numbers below the lower blocks correspond to the prey numbers in the Table 2

**Figure 6:**
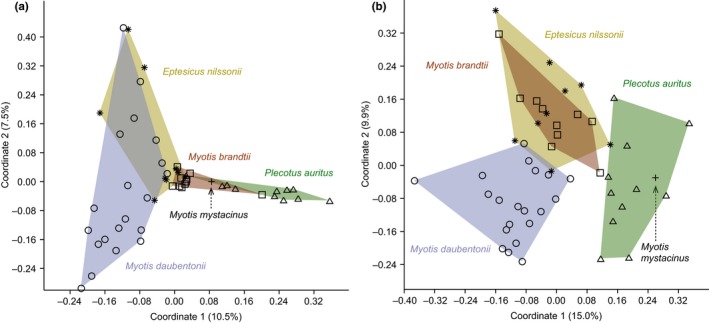
PCoA ordination based on composition of prey species in the diet of each bat species using (a) the Bray‐Curtis dissimilarity with relative reads abundances (see Methods for details) and (b) the Jaccard similarity between samples with presence/absence data in each sample. Circle = *Myotis daubentonii*; asterisk = *Eptesicus nilssonii*; square = *M. brandtii*; plus = *M. mystacinus*; and triangle = *Plecotus auritus*

The quantitative prey assemblages (RRA) seem to be very different for all the bat species, as revealed by the bipartite analysis (Figure 4a). However, when using frequencies (MFO), these patterns are not that clear (Figure 4b). In the current study, different bat species were mainly sampled in different roosts, but luckily prey use does not seem to be vastly related to the roost site, as can be seen from the bipartite analysis from the two sites where two different bat species were sampled from the same roost (Figure 5a,b). The prey use patterns were further illustrated in the PCoA ordinations: Both RRA and presence/absence data ordinations grouped the bat species according to their respective feeding guilds based on differences in the prey species assemblages (Figure 6a,b). In the RRA plotting, first coordinate explained 10.5% and the second coordinate 7.5% of the variation in the data (Figure 6a), and in the plot using presence/absence data, the first and the second coordinates explained 15% and 9.9% of the variation (Figure 6b), respectively, so for both data types a large part of the variation remained unexplained. Altogether 44 common prey species were shared by all the bat species, and 90 more equally common prey species were shared by four bat species (Table 2; Silvonen, Top‐Jensen, & Fibiger, 2014).

### 3.3 Dietary patterns in the feeding guilds

The feeding guilds are also easily separated by looking the diet at the prey family level (here using percentages from relative read abundance data, but approximately the same ratios can be drawn from the presence–absence data; Table 2): The trawling species (*M. daubentonii*) predominantly consumes a single prey family, Chironomidae (45.8% of all the reads), which is a highly abundant and species‐rich family in southwestern Finland (Lilley, Ruokolainen, Vesterinen, Paasivirta, & Norrdahl, 2012; Paasivirta, 2012, 2014 ), but constrained to the vicinity of aquatic environment, where the bat collects its prey from the water surface (Nilsson, 1997). The gleaner (*P. auritus*) relies on the plentiful moth family Noctuidae (57.2%), which is either caught in flight or from surfaces on vegetation, as some of the prey species are mainly diurnal (Silvonen et al., 2014). The other largely consumed prey family for *P. auritus* was the coleopteran family Carabidae (18.7%), which is most probably foraged from the ground. The third guild, hawkers, consists of three bat species (*E. nilssonii, M. brandtii,* and *M. mystacinus*), which all have distinct prey family spectrum. *E. nilssonii* is known to be Nematocera specialist (Rydell, 1986), and we can confirm this observation: *E. nilssonii* preyed upon Pediciidae (21.3%), Trichoceridae (18.4%), Tipulidae (13.0%), and also on chironomids (10.7%). The other two hawkers relied solely on moths: *M. brandtii*'s menu included Tortricidae (26.5%) and Geometridae (24.3%). Interestingly, at least one very abundant prey species *Agriopis aurantiaria* (Geometridae) only flies during October and after that, so this moth must have been caught by *M. brandtii* as larvae on leafs or while hanging from the tree (Silvonen et al., 2014). On the other hand, *M. mystacinus* foraged on the moth families Argyresthiidae (21.0%), Geometridae (16.5%), and Lypusidae (11.3%), which all have distinct life strategies and behavioral ecologies (Silvonen et al., 2014).

### 3.4 Temporal aspects and predator‐prey size analysis

The strong assorting patterns of different bat species seen in plotwebs and PCoA were confirmed when comparing all bat species’ diet's together in the analysis of variance (Table 3: Predator: RRA data, *df* = 4, *R* ^2^ = 0.12, *p* = 0.0001; PA data, *df* = 4, *R* ^2^ = 0.05, *p* = 0.0033). Despite the limited temporal span of the sampling for each bat (Table 1: 8 weeks for *M. daubentonii* and *P. auritus*; 5 weeks for *M. brandtii* and *E. nilssonii*), we tested the dietary variation in time, but found no significant variation between weeks (Table 3: Week). Temporal pattern was same for all bat species (Table 3: Predator × Week).

**Table 3:** Permutational multivariate analysis of variance (adonis) for prey communities for the studied bat species using Bray–Curtis dissimilarity matrix (for RRA) or Jaccard similarity (for presence–absence data) of presence or absence of prey species in each sample. Terms added sequentially (first to last) to the model. The only significant Bonferroni‐corrected *p*‐value (*p*_b_) is denoted with an asterisk, indicating that as a whole, the diet changes during the sampling season, although this effect was only observed with the PA data, but not in the RRA data

Predictor	*df*	*F*	*R* ^2^	*p*_b_
Relative read abundance data				
Predator	4	1.46	0.12	0.0001*
Week	10	0.92	0.18	0.9544
Predator × Week	7	0.96	0.13	0.7598
Residuals	29		0.57	
Total	50		1.00	
Presence/absence data				
Predator	4	1.77	0.13	0.0001*
Week	10	1.06	0.20	0.1372
Predator × Week	7	0.99	0.13	0.5561
Residuals	29		0.54	
Total	50		1.00	

When the prey assemblages were analyzed separately in pairwise PERMANOVA between species, the diet was significantly different in all compared pairs, except those with *M. mystacinus*, which was present in the sample with only one sample (Table 4). The same pattern occurred in both RRA and PA data (Table 4). The diet explained only 6%–13% of the total variance (Table 4).

**Table 4:** Pairwise permutational multivariate analysis of variance (pairwise.adonis) for prey communities for each of the studied bat species using Bray–Curtis dissimilarity matrix (for RRA) or Jaccard similarity (for presence–absence data) of presence or absence of prey species in each sample. Significant Bonferroni‐corrected *p*‐values (*p*_b_) are denoted with an asterisk. All the bat species pairs significantly differ in their prey species composition, except comparisons with *M. mystacinus*, which was represented with only one sample

Pairs	*df*	*F*	*R* ^2^	*p*_b_
Relative read abundance data				
*Plecotus auritus* versus* Myotis mystacinus*	11	1.29	0.11	1.00
*P. auritus* versus* M. daubentonii*	30	3.07	0.10	0.01^*^
*P. auritus* versus* M. brandtii*	20	2.35	0.11	0.01^*^
*P. auritus* versus* Eptesicus nilssonii*	19	2.34	0.12	0.01^*^
*M. mystacinus* versus* M. daubentonii*	20	1.19	0.06	0.49
*M. mystacinus* versus* M. brandtii*	10	1.03	0.10	1.00
*M. mystacinus* versus* E. nilssonii*	9	1.10	0.12	1.00
*M. daubentonii* versus* M. brandtii*	29	2.24	0.07	0.01^*^
*M. daubentonii* versus* E. nilssonii*	28	1.60	0.06	0.05^*^
*M. brandtii* versus* E. nilssonii*	18	1.59	0.09	0.04^*^
Presence/absence data				
*P. auritus* versus* M. mystacinus*	11	1.16	0.10	1.00
*P. auritus* versus* M. daubentonii*	30	3.83	0.12	0.01^*^
*P. auritus* versus* M. brandtii*	20	2.81	0.13	0.01^*^
*P. auritus* versus* E. nilssonii*	19	2.52	0.12	0.01^*^
*M. mystacinus* versus* M. daubentonii*	20	1.44	0.07	1.00
*M. mystacinus* versus* M. brandtii*	10	1.21	0.12	0.88
*M. mystacinus* versus* E. nilssonii*	9	1.22	0.13	1.00
*M. daubentonii* versus* M. brandtii*	29	2.55	0.08	0.01^*^
*M. daubentonii* versus* E. nilssonii*	28	2.63	0.09	0.01^*^
*M. brandtii* versus* E. nilssonii*	18	1.65	0.09	0.01^*^

**Figure 7:**
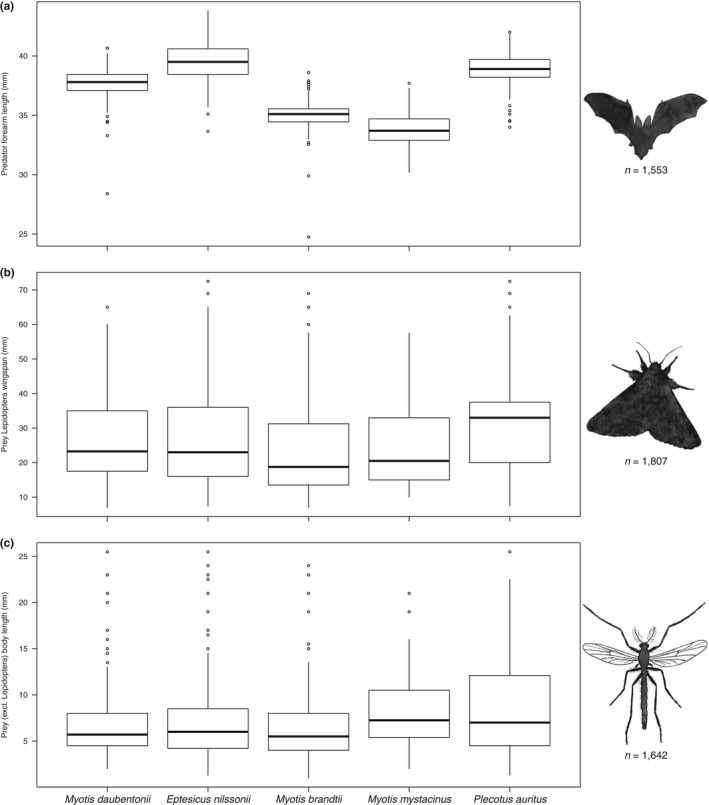
(a) Size of adult bats (measured by the length of forearm), (b) size of lepidopteran prey taxa (measured by the wingspan), and (c) size of other than lepidopteran prey taxa (measured by the body length) for each of bat species in the current study. The number of records is denoted for each group

The bat species differed significantly in size according to the banding data (Figure 7a, Kruskal–Wallis H = 867.29, *df* = 4, *p* < 0.0001), further confirmed by the pairwise analysis, where all the bats differed from each other significantly (Table 5). Similarly, the prey size differed significantly between bat species (Lepidoptera prey: H = 118.58, *df* = 4, *p* < 0.0001; other prey H = 34.5, *df* = 4, *p* < 0.0001). The pairwise analysis indicated that the diet of *P. auritus* consisted of lepidopteran prey of larger size than any of the other bat species. A similar, but not identical, pattern was observed for other than lepidopteran prey, in which *P. auritus* diet size was similar only to *M. mystacinus*. For *M. brandtii*, the lepidopteran prey size was significantly smaller compared to the other species, except for *M. mystacinus*, but other prey taxa differed in size with *P. auritus* only (Table 6). On average, *P. auritus* consumed the largest prey (Figure 7b,c; Table 6), whereas *M. brandtii* consumed the smallest prey (Figure 7b,c; Table 6).

**Table 5:** Tukey and Kramer (Nemenyi) test with Tukey‐Dist approximation for independent samples with R package “PMCMR” between all the bat species for bat forearm length, Lepidoptera prey wing span, or other prey body length. The number of records is listed for each group. The significant *p*‐values are bolded (chi‐square was corrected for ties)

Compared pairs	Bats n = 1,553 pchisq	Lepidoptera n = 1,807 pchisq	Other prey n = 1,642 pchisq
*Plecotus auritus* versus* Myotis mystacinus*	**<0.0001**	**0.0008**	0.9980
*P. auritus* versus* M. daubentonii*	**<0.0001**	**<0.0001**	**<0.0001**
*P. auritus* versus* M. brandtii*	**<0.0001**	**<0.0001**	**<0.0001**
*P. auritus* versus* E. nilssonii*	0.5700	**0.0003**	**0.0040**
*M. mystacinus* versus* M. daubentonii*	**<0.0001**	0.6635	0.2240
*M. mystacinus* versus* M. brandtii*	0.4800	0.8516	0.1590
*M. mystacinus* versus* Eptesicus nilssonii*	**<0.0001**	0.7223	0.3680
*M. daubentonii* versus* M. brandtii*	**<0.0001**	**<0.0001**	0.9810
*M. daubentonii* versus* E. nilssonii*	**<0.0001**	1.0000	0.9580
*M. brandtii* versus* E. nilssonii*	**<0.0001**	**0.0010**	**<0.0001**

**Table 6:** Average sizes with standard deviations for all the bat species (bat forearm length), prey size (Lepidoptera prey wing span and for other prey body length) with standard deviations for each group

Bat species	Bats	Lepidoptera	Other prey
*Myotis daubentonii*	37.75 ± 1.03	26.12 ± 11.73	6.68 ± 3.62
*Eptesicus nilssonii*	39.49 ± 1.65	27.05 ± 14.10	7.05 ± 4.20
*M. brandtii*	35.01 ± 1.16	22.54 ± 11.99	6.62 ± 3.78
*M. mystacinus*	33.86 ± 1.34	23.86 ± 10.85	8.59 ± 4.68
*Plecotus auritus*	38.80 ± 1.57	30.85 ± 13.17	8.98 ± 5.52

## 4 DISCUSSION

Co‐occurring species with a relatively short active season offer an excellent setup for the study of dietary strategies. Here, we identified 547 prey species in the diet of five common and abundant boreal vespertilionid bat species. All species fed mainly on two insect orders (Diptera or Lepidoptera), which undoubtedly are among the most available dietary groups (with Coleoptera) in terms of species richness (Erwin, 1982; Stork, 2018) and probably for biomass, although reliable biomass estimates are lacking. The three feeding guilds (trawlers, hawkers, and gleaners) are clearly separated by diet in the data. Moreover, the dietary composition between all bat species differed significantly, a pattern that persisted throughout the results. This pattern was strong enough to be observed in all the interpretations of the molecular data (presence/absence, frequencies, and read count data analysis). The sampling week did not explain the diet for any bat species, but we found differences in average prey size consumed by the bat species, and a positive correlation between bat species size and size of prey, although with a fine marginal.

In concordance with dietary studies on insectivorous bats, we also revealed a high frequency of lepidopteran and Dipteran species in the diets of the sampled species (Clare et al., 2014; Shively et al., 2017; Vesterinen et al., 2016). In fact, combined, these two orders constitute the majority of all predation records in the whole study, regardless of the data type (read counts, frequency, or presence/absence). Especially, *P. auritus* appears to utilize lepidopteran prey species to a higher degree compared to the other species, although rather surprisingly, ~20% of the diet (in terms of relative read abundance) of *P. auritus* appears to consist of Coleoptera, particularly ground beetles. All other invertebrate orders are less relied on, although Trichoptera and Neuroptera constitute a small part of the diet in some species. This is expected, seeing as these orders include mass‐emerging species, such as *Oecetis ochraea* (Trichoptera, Leptoceridae), or species which are active and available as prey throughout the season, such as *Brachyderes incanus* (Coleoptera, Curculionidae), or otherwise very common and abundant species, such as *Chrysoperla carnea* (Neuroptera), are all found in this study (Vesterinen et al., 2013, 2016 ). This primarily highlights the huge biomass and species diversity found in Lepidoptera and Diptera, but secondly, also further establishes the importance of these orders to bat species diversity. Because of the huge biomass of insects worldwide, there are numerous predators in addition to bats, such as fish, birds and even predatory insects, consuming these, and other arthropods as their primary food source (fish: Jakubavičiūtė, Bergström, Eklöf, Haenel, & Bourlat, 2017; dragonflies: Kaunisto et al., 2017; birds, spiders: Wirta et al., 2015). Surprisingly, the prey order‐level similarity between different predator taxa is surprisingly high when comparing our results to the aforementioned studies, especially between bats and other flying insectivores.

The patterns detected in this study indicate the dominance of Diptera and Lepidoptera (Dip&Lep) in the diet of boreal bats. At first glance, this pattern could in theory be caused by the so‐called primer bias, which means that the chosen primers amplify some taxa (such as Dip&Lep) more than others (such as Coleoptera or arachnids). The primers used in this study, the most widely applied and very functional Zeale primers (Zeale et al., 2011), have received some (in vitro) criticism claiming they may over‐estimate Dip&Lep (Clarke et al., 2014). However, we feel that these two orders, Diptera and Lepidoptera, are arguably among the most species‐rich and abundant insect orders in Finland and especially in the study area (see, e.g., Supplement 1 in Vesterinen et al., 2016), and thus, the dietary patterns found by these markers seem very intuitive and logical. Furthermore, we found a large proportion of Coleoptera in the diet of *P. auritus*, suggesting that the claimed bias is not too strong to detect abundant prey outside Dip&Lep orders. At the time of conducting this study, no other primer pair has been shown to amplify a short target (to enable detection of highly fragmented prey DNA), and at the same time exclude bats, while including (mostly) all arthropod prey. This said, in future studies, other primers along Zeale primers and possibly more than one (mitochondrial) loci should be used, as no primer is totally free of bias (Alberdi, Aizpurua, Gilbert, & Bohmann, 2018; Clarke et al., 2014).

The diet of each bat species remained unchanged throughout the season. This, together with the high number of different species consumed, suggests the role of insectivorous bats as (perhaps habitat‐related) specialists (Vesterinen et al., 2016), although some opportunistic generalism has been observed (Salinas‐Ramos, Herrera Montalvo, León‐Regagnon, Arrizabalaga‐Escudero, & Clare, 2015; Vesterinen et al., 2013). This suggests that the diets of our study species could be determined by the abundance and availability of insect prey instead of any particular predator‐specific characteristic. In fact, it has previously been reported that bat diet responds to local insect population fluctuations (Aizpurua et al., 2018; Clare, Barber, Sweeney, Hebert, & Fenton, 2011; Sedlock, Krüger, & Clare, 2014; Vesterinen et al., 2016). Razgour et al. (2011) reported temporal shifts in the proportional frequencies of Lepidoptera and Diptera prey of *P. auritus*. We found no evidence of shift in these frequencies in our *P. auritus* samples. At the latitude where our study was conducted, there is only a two‐ to four‐week difference between the highest abundance peaks for Diptera and Lepidoptera, and furthermore, it may be that even during the period of low abundance, there are still more than enough prey items available for bats (Vesterinen et al., 2016).

Diet comparisons between sympatric bat species using molecular methods are still relatively scarce, but often show considerable overlap in diet, even at the lower taxon level (Krüger, Clare, Greif, et al., 2014; Krüger, Clare, Symondson, et al., 2014; Salinas‐Ramos et al., 2015; Ware, 2016). Most studies focus on either closely related species, or species which share a feeding guild, such as the two trawling bats (*M. daubentonii* and *M. dasycneme*) in a study by Krüger, Clare, Greif, et al., 2014; Krüger, Clare, Symondson, et al., 2014. In the current study, we compared the diet of five vespertilionid bats, representing three different guilds. According to our analysis, all three guilds are clearly evident, with little overlap between the aerial hawkers (*M. brandtii* and *E. nilssonii*) and the trawling bat (*M. daubentonii*). These dietary overlaps are likely to be explained by the opportunistic and sporadic consumption of a very few prey items, such as mass‐emerging chironomids, moths, mayflies, and caddisflies. *Plecotus auritus*, the species considered a gleaner and moth specialist, showed a marked difference in PCoA ordination compared to the other two groups. We also discovered a significant difference in the size of prey consumed, with the larger *P. auritus* consuming larger prey species, whereas the smaller bat, *M. brandtii*, consumed smaller prey items. This is not surprising as it is generally accepted that the echolocation used by aerial insectivorous bats renders smaller prey items unavailable to larger bats (Brigham, 1991; Waters, Rydell, & Jones, 1995). Additionally, *P. auritus,* among other members of the genus, possesses a suite of morphological characters (low wing‐loading, large pinna, low‐frequency hearing), which allow them to use both acoustic gleaning and aerial‐hawking foraging strategies to capture prey (Coles, Guppy, Anderson, & Schlegel, 1989; Norberg & Rayner, 1987). It is possible that some noctuid prey individuals have been foraged as larvae, as the flight peak of most noctuid prey in the current study is later than the sampling period.(Finnish Biodiversity Information Facility/FinBIF. http://https://tun.fi/HBF.31668; accessed 2018‐08‐26). These strategies permit the genus to occupy a specialized feeding niche within European bat assemblages (Roswag et al., 2018).

Interestingly, the two aerial‐hawking species studied here, *E. nilssonii* and *M. brandtii*, showed considerable overlap in diet according to the PCoA, analogous to *M. dasycneme* and *M. daubentonii* (Krüger, Harms, Fichtner, Wolz, & Sommer, 2012), despite representing two different genera. Taking a closer look at the diets of the two species, we notice that regardless of both species relying heavily on Lepidoptera and Diptera, the proportions of these taxa in the diets differ considerably and the diets consist of entirely different prey families. Whereas the majority of the *E. nilssonii* diet consists of nematoceran Diptera (>60% of reads are either Pediciidae, Trichoceridae, Tipulidae, or Chironomidae for *E. nilssonii*), the *M. brandtii* diet reveals a greater proportion of Lepidoptera (>50% of reads are Geometridae and Tortricidae for *M. brandtii*). In addition to this, the lepidopteran diet consumed by *M. brandtii* is considerably smaller in size compared to *E. nilssonii*. These finer scale differences in the diet of these two aerial‐hawking species could be explained by differences in other dimensions of their respective ecological niches. For instance, *E. nilssonii* forages in relatively open spaces (forest edges, clearings, open gardens, etc.), whereas *M. brandtii* prefers more confined spaces with forest cover (Dietz, Nill, & Helversen, 2009). This is resource partitioning that could be further dissected by looking at isotopic niches, for instance, to give a complementary scenery to dietary ecology besides DNA‐based analysis (Schmidt, Mosbacher, Vesterinen, Roslin, & Michelsen, 2018). Another option would be to increase sampling effort to obtain an even more robust overview of the main prey items. Information on the identified major dietary taxa could then be used to deduct the main foraging habitat, as presented by Alberdi, Garin, Aizpurua, and Aihartza (2012).

The molecular work carried out in this analysis not only highlights the deep insight offered by metabarcoding, but also underlines the dynamic and complementary nature of DNA‐based analysis. Based on our earlier field work, we had chosen species‐specific roosting sites for the diet analysis of five bat species, to obtain an equal sampling effort. However, when confirming the fecal “donor” by the means of metabarcoding, we noticed some discrepancies between the field data and confirmed data, that is, our *M. mystacinus* roost was confirmed as an *E. nilssonii* roost. In future, the molecular confirmation of noninvasively collected samples should be a standard approach, either by traditional Sanger sequencing or cost‐effective next‐generation sequencing (NGS), depending on the number of samples and the predator and prey species. Also, the importance of a comprehensive reference library (Mutanen et al., 2012; Pentinsaari, Hebert, & Mutanen, 2014; Pilipenko, Salmela, & Vesterinen, 2012), which allows the correct and reliable identification of most prey items, needs to be pointed out once more. This offers the possibility of deeper ecological dietary studies, such as prey size analysis (Pentinsaari et al., 2014). While some prey items had not been described with a scientific species‐level name in this study, a reliable estimate of their size could be inferred using the so‐called barcode index numbers (BIN; Ratnasingham & Hebert, 2013) to trace the images for measurements. This emphasizes the significance of public and easy‐accessible reference library systems, such as BOLD (Ratnasingham & Hebert, 2007). Although some studies still rely on OTUs (operational taxonomical units) instead of biological species, we highlight the importance of actual prey species determination, which allows a deeper and more robust insight into dietary ecology.

The main drawbacks of the molecular methods are the highly challenging interpretations of the quantitative aspects of the diet, that is, are the most frequently consumed prey items also the most important in terms of biomass and energy gain? While the current practice in many molecular ecological dietary studies using metabarcoding appears to mostly rely on frequency of occurrence (but see Vesterinen et al., 2016), the read counts may actually hold some important quantitative information (Deagle et al., 2018). Here, we tested our data using both frequency of occurrence and read count data and found no major differences in the outcome of the analysis, or more importantly, in the interpretation of the results. This suggests our data have strong ecological message that holds despite the methodological approach used.

Our study supports the existence of dietary flexibility in generalist bats and dietary niche overlapping, especially in bats of the same feeding guild in a highly seasonal ecosystem (Roswag et al., 2018). In fact, it could be the flexibility in feeding strategies which allows species to sustain populations in arctic and subarctic regions (Shively et al., 2017). Additionally, a great proportion of niche differentiation most likely also occurs outside the diet dimension where an almost infinite number of possible axes exist for competing species in the *n*‐dimensional niche hyper‐volume (Hutchinson, 1957). Even minor differences in a number of different axes can result in a substantial overall difference (Privitera et al., 2008). Clearly, the next logical step is to utilize deep dietary analysis, alongside other ecological (LIDAR: light detection and ranging method, etc.) and behavioral (GPS‐tracking) datasets to begin to understand niche realization and resource partitioning in species to a far higher accuracy than has been available to date.

## AUTHOR CONTRIBUTIONS

EJV and TML designed the study, collected the data, and wrote the first version of manuscript. ASB collected samples in the field and gathered prey species measurements and the map data. AIEP and EJV conducted the molecular work and data analysis. All authors contributed to the final version of the manuscript.

## DATA ACCESSIBILITY

Labeled raw reads and OTUs are available in the Dryad Digital Repository: http://https://doi.org/10.5061/dryad.6880rf1.
